# 

**Published:** 1890-07

**Authors:** 


### Society Meetings.

The American Society of Microscopists will hold its next annual meeting in Detroit, Mich., during Tuesday, Wednesday, Thursday and Friday, August 12, 13, 14, and 15, 1890, under the presidency of Dr. George E. Fell, of Buffalo.

The Meeting of Railway Surgeons.—The annual meeting of the National Association of Railway Surgeons, after a most profitable meeting at Kansas City, adjourned on May 2d, to meet at Buffalo, N. Y., May 2, 1891. The following are the officers for the ensuing year: President, Dr. Warren B. Outten, St. Louis; First Vice-President, Dr. S. S. Thorn, Toledo; Corresponding Secretary, Dr. A. G. Gumaer, Buffalo; Recording Secretary, Dr. E. R. Lewis, Kansas City; Treasurer, Dr. R. Harvey Reed, Mansfield, Ohio.—*St. Louis Medical and Surgical Journal.*

---

The American Association of Obstetricians and Gynecologists will hold its next annual meeting in Philadelphia, Tuesday, Wednesday, and Thursday, September 16, 17, and 18, 1890, under the presidency of Dr. E. E. Montgomery, of Philadelphia, and in the hall of the College of Physicians.

---

The Mississippi Valley Medical Association will hold its annual meeting in Louisville, Ky, October 8–11, 1890, under the presidency of Dr. Joseph M. Mathews of that city. This popular organization may look forward to one of its most prosperous meetings under such favorable auspices.

### BOOKS AND PAMPHLETS RECEIVED.

Formulaire Aide-Mémoire De La Faculté De Médecine et des Médecius Des Hopitaux De Paris. Par Dr. Fernand Roux. Mention Honorable D L'Institute, etc., etc. Paris: G. Stembeil, Editeur. 1890.

University Medical College of Kansas City. Third Annual Announcement and Catalogue of Session, 1889-90.

Uric Acid Diathesis in Affections of the Eye, Ear, Throat, and Nose. By W. Cheatham, M. D., Louisville, Kentucky. Reprint.

Intestinal Anastomotic Operations with Segmented Rubber Rings, with some practical suggestions as to their use in other surgical operations. By A. V. L. Brokaw, M. D., St. Louis, Mo. Reprint.

New Method of Performing Pylorectomy, with remarks upon Intestinal Anastomotic Operations. By A. V. L. Brokaw, M. D., St. Louis, Mo. Reprint.

Health in Michigan. Report for May, 1890.

Communication from Jack the Ripper. Reprint from the *Medical Mirror*. St. Louis, May, 1890.

Apparent Cancerous Transformation of Syphiloma of the Tongue. Excision of the Tongue by the Galvanic-Cautery. By G. Frank Lydston, M. D., Chicago, Ill. Reprint.

A Description of Cochran's Method for the Determination of Fat in Milk, for the use of Dairymen. Bulletin of the Agricultural Experiment Station, Chemical Division, of Cornell University, College of Agriculture.

Abstract of Sanitary Reports, Vol. V., Nos. 23, 24, 25 and 26. Marine Hospital Bureau, Washington, D. C.

A Consideration of Sexual Neurasthenia. By Bransford Lewis, M. D., St. Louis, Mo. Reprint.

Fifth Annual Report of the Board of Health, the City of Newport, R. I., for the year 1889.

Fever: Thermotaxis and Calorimetry of Malarial Fever. By Isaac Ott, M. D., ex-Fellow in Biology, John Hopkins University. E. D. Vogel, Bookseller, Easton, Pa.

The Brooklyn Health Exhibition, held under the auspices of the Local Committee of Arrangements of the American Public Health Association, Brooklyn, N. Y., October 22 to November 30, 1889. Reprint.

Chloralamide as a Hypnotic. By W. Hale White, M. D., F. R. C. P., London. Reprint.

Fishing and Hunting Resorts of the Grand Trunk Railway. Season 1890.

Speech of the Hon. John P. Jones, of Nevada, on the Free Coinage of Silver, in the U. S. Senate, May 12 and 13, 1890. Reprint.

